# Long-Term Stability of Two Thermoplastic Polymers Modified with Silver Nanoparticles

**DOI:** 10.3390/nano9010061

**Published:** 2019-01-04

**Authors:** Magdalena Ziąbka, Michał Dziadek

**Affiliations:** 1 AGH University of Science and Technology, Faculty of Materials Science and Ceramics, Department of Ceramics and Refractories, 30-059 Krakow, Poland; 2 AGH University of Science and Technology, Faculty of Materials Science and Ceramics, Department of Glass Technology and Amorphous Coatings, 30-059 Krakow, Poland; dziadek@agh.edu.pl

**Keywords:** biostable polymers, silver nanoparticles, biomaterials

## Abstract

The aim of this study was to investigate the mechanical properties of polymeric composites prepared via extrusion and injection moulding. Four stable thermoplastic polymers were used as composites matrices (two kinds of polymethyl methacrylate and two kinds of co-polymer acrylonitrile-butadiene-styrene). Silver nanoparticles AgNPs were used as a modifying phase. Mechanical properties of testes materials were determined during the uniaxial stretching. Surface properties such as roughness and contact angle were also evaluated. The materials’ stability was assessed using scanning electron microscopy and non-destructive ultrasonic testing. All measurements were carried out at time intervals, determining both the initial parameters and after 6 and 12 months of incubation in deionized water. The obtained results proved that neither the preparation technology nor the amount of the modifier adversely affect the mechanical properties of the tested composites. The incorporated modifier does not change the surface properties significantly. The studies conducted after the materials’ incubation in water indicate their stability.

## 1. Introduction

A wide variety of polymers is applied in medicine as biomaterials. Biostable polymers are commonly used for orthopedic applications, in dentistry and otolaryngology, for vascular grafts, surgical meshes, ligaments, tendon repairs, balloons of catheters, dialysis membranes and in the plastic surgery [1]. Their main advantage is the lack of degradation in the human body’s environment. Additionally, polymers show high resistance to the biologically active in vivo environment and sustain their physicochemical properties—in particular the mechanical ones—on a constant level as a function of time. What is more, physiological interactions of biostable materials do not lead to the alterations in their properties or the loss of functionality during the service life of biomedical polymers [2]. The most popular biostable synthetic polymers are polyoefines (polyethylene—PE, high density polyethylene—HDPE, ultrahigh molecular weight polyethylene—UHMWPE, polypropylene—PP), polytetrafluoroethylene—PTFE, polyamide—PA, polyether polyurethane elastomers—PEU, silicones, polycarbonates—PC, polyethylene terephthalate—PET, acrylic polymers (polymethyl methacrylate—PMMA), acrylonitrile butadiene styrene—ABS and polyetheretherketone—PEEK.

Among polyolefines UHMWPE is the most widely used e.g., for orthopedic implants, such as joint prostheses [3]. High density polyethylene and polypropylene are present in medical care and in public health. They are used for middle ear prostheses [4], long-term catheters, acetabular endoprostheses, facial restoration, nasal dorsal augmentation, mandibular contours, orbital floor and socket reconstruction and augmentation of facial contours [5]. They serve as materials to produce connectors, tees and couplers for pipes and drains, straight and angled taps, plugs, stoppers, plastic gloves, dishes, syringes, intravascular catheters and meshes for vaginal repair of anterior vaginal wall sutures [6,7].

Another polymer widely used in medicine is polytetrafluoroethylene—PTFE (teflon). It is mainly applied to obtain vascular graft, surgical meshes, ligaments, tendon repair and middle ear implants—partial or total ossicular replacement prostheses [8,9,10].

Polyether polyurethane (PEU) elastomers are yet other types of biostable and biocompatible polymers used to replace silicone rubber as pacemaker surface coatings. The problem arises as biostability of these polymers does not meet long term standards, despite the promising initial results. Nevertheless, PEUs can still be used to obtain vascular prostheses, blood pumps, intravascular balloons, breast, esophagus and trachea implants, fallopian tubes and blood dialysis membranes are made of them. They are also used for the production of sutures and ligaments as well as dressings, catheters, blood storing bags and elements of artificial heart [11,12,13,14].

In medical devices and pharmaceutical applications, silicones are applied because of their biocompatibility in various physical forms. Nowadays they are used in numerous life-saving medical devices, such as pacemakers or hydrocephalic shunts [15], in plastic surgery for covers of breast implants, artificial joints of phalanges, urological catheters, arteriovenous fistulas, tendon prostheses and for drains in laryngology [16].

Polycarbonates—PCs—belong to the engineering thermoplastic materials widely tested and commonly used in the industry of medical devices. They are applied to produce artificial heart chambers as well as rings and handles which are elements of artificial heart valves, dialysis membranes and containers and optical and contact lenses [17,18]. Polycarbonates offer an unusual combination of strength, rigidity and toughness that helps to prevent potentially life-threatening material failures. In addition, they are characterized by glasslike clarity—a critical factor for clinical and diagnostic settings where visibility of tissues, blood and other fluids is required.

Another interesting biostable polymer is methyl methacrylate—PMMA. Due to its high rigidity it is useful in dentistry and orthopedics. PMMA is used to obtain bone cements, contact and intraocular lenses, screw fixations in bones, as a filler for bone cavities and skull defects, as vertebrae stabilization in osteoporotic patients and for dialysis membranes [19].

In general, polyamides are increasingly applied to produce medical devices, e.g., implants, fibers, suture materials or functional surfaces thanks to the high tensile strength and resistance to solvents, oils and body fluids and their high biocompatibility. However, polymers’ low hydrophilicity and high crystallinity often require additional modifications [20].

Acrylonitrile butadiene styrene resins are advantageous in medical industry due to the exceptional purity, low residual monomers, lot-to-lot consistency and superior whiteness. They have been long valued for their durability, toughness and aesthetic appeal. ABS polymer is also a very promising material for a prototype of the middle ear implant [21,22,23]. Besides being commonly used for infusions systems, such as connectors, cups, spikes and roller clamps, it is also used for respiratory, auto injection and other medical devices [24].

Polyetheretherketone (PEEK) is a polyaromatic semi-crystalline thermoplastic polymer with mechanical properties favourable for biomedical applications. It is considered a hard and stable polymer for orthopedic applications or for inner lining of catheters. The carbon reinforced PEEK is applied in spine surgery for fusion cages [25,26].

Although such a variety of biostable polymers is already present on the medical market, scientists are constantly working on polymer modifications to improve their physicochemical and biological properties. It is known that incorporation of different nanofillers into polymer matrices may improve the composite materials. Bioactive additives, such as hydroxyapatite—HA, tri-calcium-phosphate—β-TCP or bioglass facilitate the bone-implant interaction [27,28,29]. Metals in a form of silver, gold, copper and titanium dioxide nanoparticles show strong antibacterial activity [30,31,32,33]. The advantage of our composites compared to other polymers and composites used in medicine is an effective and long-lasting bactericidal effect in the environment of the replaced tissues. Our material has been designed for otolaryngological purposes. In middle ear surgery, there are no prostheses that effectively eliminate bacteria and fungi during otitis media. Currently, the most commonly used prostheses are made of titanium, teflon and hydroxyapatite [34,35]. However, long-term studies for hydroxyapatite prostheses have reported an extrusion rate of 5–15% [36]. Present evidence indicates that titanium transmits sound better than the heavier HA [37] but none of these implants possess bactericidal properties. The thermoplastic polymers (ABS and PMMA) make the proposed middle ear prosthesis lightweight and adjustable in terms of length. Moreover, silver nanoparticles provide a long-lasting bactericidal effect of nanocomposite prosthesis, which was proved in our previous works [22,23].

Nanocomposite materials composed of polymers with incorporated metallic nanoparticles are endowed with distinct optical, electrical and catalytic properties with high applicability in the fields of catalysis, bioengineering, photonics and electronics [38]. However, additives may also adversely affect the stability of polymers. As nanoparticles tend to agglomerate, they may lead to the degradation or decrease in mechanical properties. Taking all the above into consideration, the goal of this work was to investigate long-term stability of composite materials modified with silver nanoparticles.

## 2. Materials and Methods

Polymeric materials and composites were obtained via the process of extrusion and injection moulding. Commercially available copolymers of acrylonitrile butadiene styrene (ABS- NovodurHDM203FC and NovodurHDM205FC) and polymetacrylan methylene (PMMA-SG7 and SG10) were used as polymer matrices. Silver nanoparticles AgNPs (NanoAmor company, Katy, TX, USA) with a purity of 99.9%, 80nm particle size and density of 10.49g/cm^3^ were applied as a modifying additive. The silver participation in the various compositions was respectively 0.5% and 1% by weight. In order to obtain the oar-shaped samples the multi-staged process took place. First, the granulate was prepared and dried in the laboratory dryer at 80 °C for 6 hours. Next, the silver nanoparticles were incorporated and homogenized with polymer granules in the plasticizing chamber, using a 0.8 m-long screw. The process of homogenization was carried out in a dual cycle and the compositions were melted twice. Subsequently, the material was injected into a steel moulding form, cooled and extracted. The injection parameters were selected and adapted for the process according to the data sheet of polymer manufacturer (injection temperature in three zones was 240 °C, injection pressure—80 kg cm^−2^, flow—80%).

All the prepared samples were investigated before the incubation and after 6 and 12 months of incubation in deionized water at the temperature of 37 ± 1 °C.

The arithmetical mean roughness (Ra) of the surface was evaluated using a contact profilometer HOMMEL-ETAMIC T1000 wave (Jenoptik AG, Jena, Germany). The surface wettability was evaluated by means of the static water contact angle measurements. The static water contact angle was determined by the sessile drop method with an automatic drop shape analysis system DSA 10 Mk2 (Kruss, Hamburg, Germany). The UHQ-water droplets of 0.25 µL were applied on each pure and dry sample. The measurements were carried out in stable conditions, considering the temperature and humidity. The surface roughness parameter and static water contact angle were calculated as an average of 10 measurements and were expressed as a mean ± standard deviation (SD).

The Nova Nano SEM FEG-200 scanning electron microscope (FEI, Eindhoven, Netherlands) was used to perform a detailed examination of the samples’ microstructure. The measurements and observations were conducted in low vacuum conditions, with a secondary electron detector (SE) and the accelerated voltage of 5–18 kV. The microstructure observations were conducted on the surface and on cross-sections of samples before and after incubation. All the she samples were coated with a carbon layer.

Tensile strength (σ_M_) and Young’s modulus (Et) were determined using a universal testing machine Inspect Table Blue 5 kN with 5 kN load cell (Hegewald&Peschke, Nossen, Germany). The pre-load force was 1N, the test speed was 50 mm min^−1^. The samples for measurements were prepared according to the EN ISO 527-1 norm. The mechanical parameters were averaged from ten measurements and expressed as a mean ± standard deviation (SD).

The ultrasound velocities were measured by means of an ultrasonic pulse velocity method using an ultrasonic apparatus: the MT541 device equipped with 1 MHz transducers for longitudinal waves. The test focused on measuring the velocity of the ultrasonic wave propagating in the longitudinal direction through the oar-shaped samples intended for mechanical tests.

## 3. Results and Discussion

**Figure 1:**
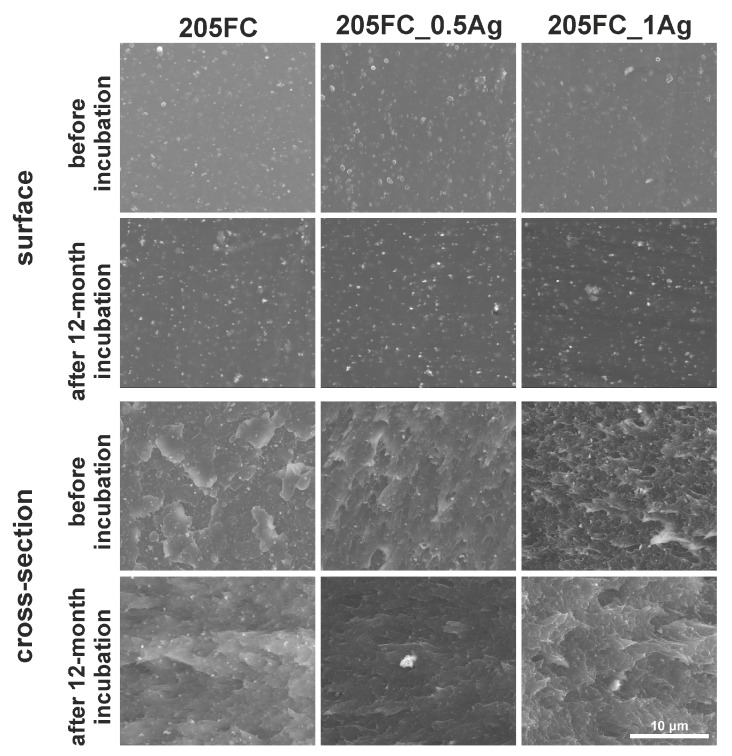
SEM images of surface and cross-section of the 205FC-based materials before and after 12-month incubation in deionized water.

**Figure 2:**
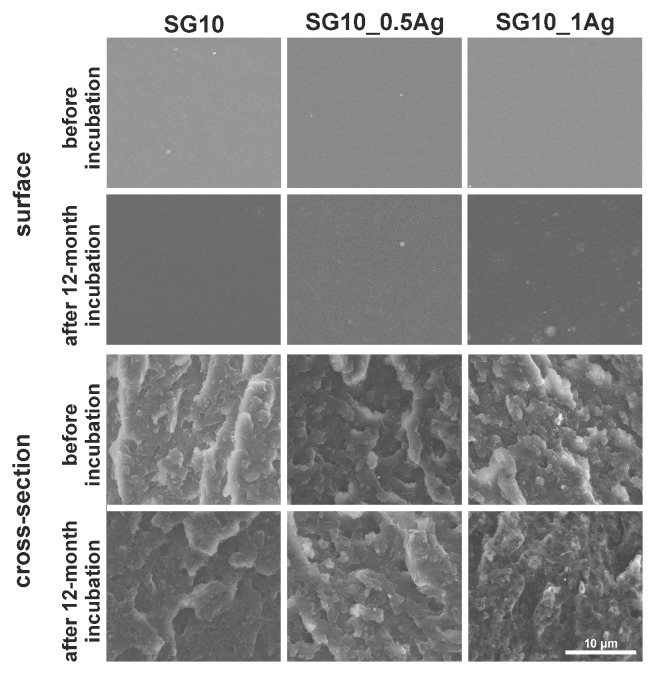
SEM images of surface and cross-section of the SG10-based materials before and after 12-month incubation in deionized water.

**Figure 3:**
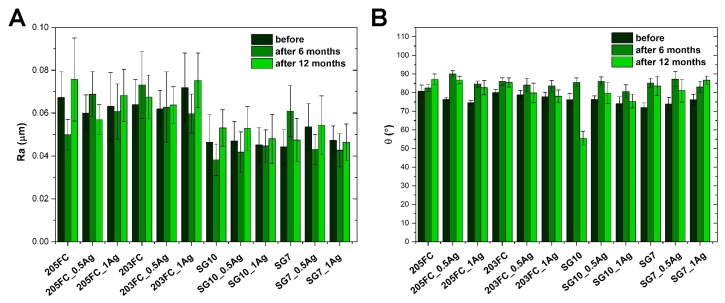
The arithmetical mean roughness (**A**) and static water contact angle (**B**) of the materials before and after 6-month and 12-month incubation in deionized water.

On the basis of microscopic observations (Figure 1 and Figure 2 and Figures S1 and S2 in Supplementary file), it was found that the surface of materials with ABS matrix (205FC and 203FC) is characterized by a higher surface development, as compared to PMMA materials (SG10 and SG7). These observations correlate with the values of the mean arithmetic profile deviation from the mean line Ra (Figure 3A). This phenomenon is related to the presence of TiO_2_ nanoparticles, occurring in both the 205FC and 203FC polymer. These nanoparticles have been used by the polymer producer as a pigment. Both groups of materials are characterized by high homogeneity of the surface. In addition, on the surface of some materials—in particular the 203FC-based polymer—there are visible cracks resulting from the impression made by the injection mould. Agglomerates of silver nanoparticles are to be observed both on the surface and cross-sections of the composite materials. The images of cross-sections also revealed the presence of small pores which are not observed on the surface of materials. The long-term incubation of materials in deionized water did not cause any noticeable changes in their microstructure, as it was evaluated for both the surface and cross-sections of the materials.

The surface roughness of materials both before and after the incubation periods in deionized water was evaluated on the basis of the Ra parameter (Figure 3A). The materials with PMMA polymer-based matrix (SG10 and SG7) were characterized by a significantly lower surface roughness in comparison to the ones based on ABS polymer (205FC and 203FC). The presence of silver nanoparticles in both types of matrix does not significantly affect the surface roughness. Before incubation, the Ra parameter for the PMMA material group ranges from 0.060–0.072 μm, while for ABS-based materials the values are in the range 0.044–0.054 μm, which indicates the low surface roughness of both groups of materials. The values of the Ra parameter after incubation in deionized water show an upward trend, which is particularly noticeable for most materials after a longer incubation period. After 12 months of incubation in deionized water, the parameter does not exceed 0.095 μm for ABS materials and 0.068 μm for PMMA materials after 12 months of incubation in deionized water.

The parameter having a significant impact on the biological response is the roughness and topography of the biomaterial surface. On the one hand, the increased surface roughness may promote bacterial adhesion and thus the development of disadvantageous bacterial biofilm. The solution to this problem may be the antibacterial effect of Ag/TiO_2_ nanoparticles. On the other hand, the incorporation of a nanofiller into the polymer matrix can improve the adhesion of osteoblasts to the surface of the material by modifying topography and/or increasing surface roughness on a nanometric scale. In addition, numerous studies indicate that the nanometric scale of the material surface can significantly regulate the behaviour of osteoblasts and osteogenic cells, affecting proliferation, intercellular signalling, gene expression and differentiation [39].

On the basis of the obtained results, the wettability of the surface of the tested materials was determined (Figure 3B). The static contact angle determined before incubation in deionized water was at a similar level for all the groups of materials and did not exceed 85°, indicating the hydrophilic nature of the surface. The introduction of a silver nanoparticle modifier in both the ABS (205FC and 203FC) and PMMA (SG7 and SG10) matrix did not significantly affect the wettability of the surface. The values of the contact angle for materials incubated in deionized water showed an increasing tendency. For most materials the highest value of the contact angle was recorded after 6 months of incubation and it did not exceed 90°.

**Figure 4:**
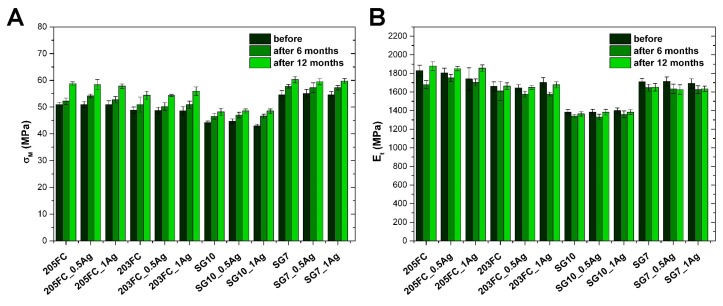
Tensile strength (**A**) and Young’s modulus (**B**) of the materials before and after 6-month and 12-month incubation in deionized water.

The tensile strength of both materials before and after 6 and 12 months of incubation in deionized water is shown in Figure 4A. Among the ABS-materials the samples based on the 203FC polymer showed slightly lower strength, as compared to the 205FC polymer-based ones. The strength value was in the range of 50–60 MPa. In the case of materials with the PMMA matrix, the ones based on the SG10 polymer were clearly lower in strength. The strength was between 45–55 MPa. In the case of polymer SG7, however, the strength was 55–60 MPa. Incorporation of silver nanoparticles did not cause significant changes in tensile strength, regardless of the type of the matrix material. For all the materials, their incubation in deionized water resulted in a 5% increase in tensile strength. Both investigated groups of polymers are amorphous, thus the toughness is determined by the motion of molecules of whole chains or their big fragments. In case of composite materials containing nanoparticles the stresses are transmitted not only by the matrix itself, but by the particles as well. The shape and size of those particles, their ability to deform and also the interaction between the matrix and the particles may cause either strengthening or weakening of the mechanical properties. The composites’ strength also depends on the amount of nanosilver, its dispersion in the matrix and adhesion in the interphase region. The increase in tensile strength observed after the incubation may be connected with formation of longer polymer chains. As a result, the mechanical properties of the material are improved [40].

Considering the values of Young’s modulus determined via a static tensile test before incubation in deionized water, a similar relationship can be observed in comparison to the tensile strength values. Namely, within the ABS group, the materials based on the 203FC polymer showed slightly lower Young’s modulus as compared to the 205FC polymer-based ones. On the other hand, among all the PMMA matrix materials, the SG10-based ones were clearly characterized by lower values of the elastic modulus. The presence of silver nanoparticles did not cause significant changes in the Young’s values. In the case of materials based on the 205FC polymer matrix, incubation in deionized water resulted in a slight decrease in the elastic modulus after 6 months. Later, the longer incubation period resulted in higher values of the tested parameter as compared to the data measured before incubation. For materials from the 203FC group and SG10 group, a slight decrease in the Young’s modulus was also observed after a 6-month incubation. Yet in this case the longer incubation resulted in returning to the value from before incubation. The Young’s modulus for SG7 materials decreased after 6-month incubation and did not change significantly after its continuation.

The tensile stress-strain graphs shown in Figure S3 (supplementary file) indicate high plasticity of the materials based on the ABS and PMMA matrices, which is confirmed by the elongated shape of the curve section after reaching the yield point. Incubation of materials in deionized water results in changes in the length of the curve section in the mentioned range, indicating at the same time changes in elongation at break. The lack of clear changes in the shape of the curve of incubated materials suggests the structural stability of the materials.

Materials intended for middle ear implants, due to their function, should be characterized by both high mechanical stability and resistance to degradation in vivo. On the one hand, their mechanical parameters should be similar to the parameters of the tissues they are meant to replace in the chain of auditory ossicles (ankles, ligaments, joints). On the other hand, they should provide effective transmission of vibrations from the tympanic membrane to the inner ear. The high in vivo stability of the material ensures stable mechanical properties, maintaining the appropriate shape of the implant, and thus enables effective transmission of vibrations during the entire lifetime.

In order to confirm the stability of mechanical parameters the propagation of the longitudinal ultrasound wave was examined.

**Figure 5:**
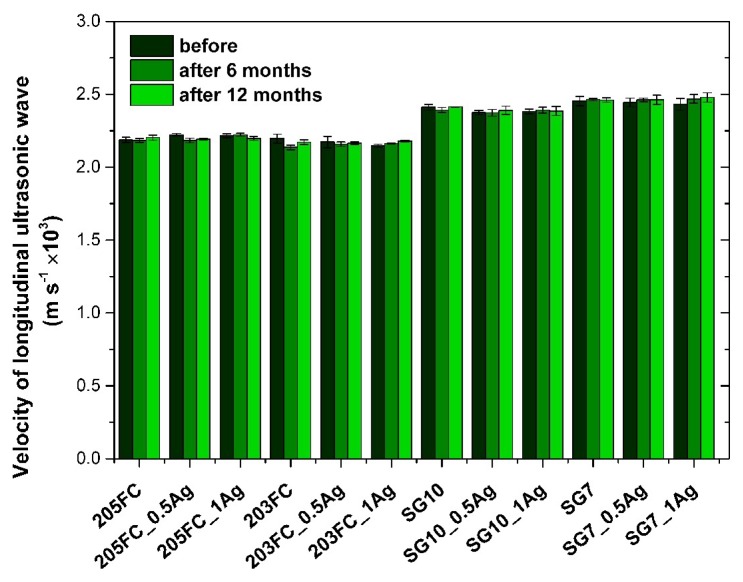
Velocity of longitudinal ultrasonic wave measured for the materials.

Figure 5 presents the values of velocity of longitudinal ultrasonic wave obtained for the materials before and after incubation in deionized water. The results indicate that the materials based on the ABS-matrix (205FC and 203FC) are characterized by significantly lower velocity of wave propagation, as compared to the materials with PMMA-matrix (SG10 and SG7). Neither the presence of the modifier in the form of silver nanoparticles nor the long-term incubation of materials in deionized water prove a significant effect on the tested parameter, regardless of the type of polymer matrix.

## 4. Conclusions

In conclusion, long-term studies conducted in vitro indicate that the composite materials based on both polymers—ABS and PMMA—show high stability of mechanical and surface properties. The presence of both concentrations of silver nanoparticles does not significantly affect the analysed parameters—microstructure, wettability and surface roughness or the tensile strength and stiffness of materials. The obtained results suggest that the nanocomposites produced in such a way can be used as stable implant materials, for example in the reconstruction of the ossicular chain.

In perspective, our results concerning stability of medical polymers modified with bioactive nanoparticles might significantly influence further research on polymers which are suitable for medical device production and are implanted in human body for longer than 30 days. Leading producers regard prospects of application of their products, depending on class of risk and time of application. Incorporation of any biologically active additives into polymers matrix results in a new classification and risk level assessment of the product. These are the main inhibitors preventing from implementation to the market new, often better products.

Outcomes of long-term research, showing biological and physicochemical properties might be a proof of safety for using composites in medicine and implantology.

## Supplementary Materials

The following are available online at http://www.mdpi.com/2079-4991/9/1/61/s1, Figure S1: SEM images of surface and cross-section of the 203FC-based materials before and after 12-month incubation in deionized water; Figure S2: SEM images of surface and cross-section of the SG7-based materials before and 12-month incubation in deionized water; Figure S3: Tensile stress-strain curve of the 205FC- (**A**), 203FC- (B), SG10- (**C**), and SG7-based (**D**) materials before and after 6-month and 12-month incubation in deionized water.
