# Supplementary material for: Long-Term Stability of Two Thermoplastic Polymers Modified with Silver Nanoparticles
Source: Nanomaterials (Basel). 2019 Jan 4;9(1):61. doi: 10.3390/nano9010061 (PMC6359626; doi:10.3390/nano9010061)
Supplement: Supplementary file 1 [file nanomaterials-09-00061-s001.pdf]

Supplementary Materials

Long-term stability of two thermoplastic polymers modified with silver nanoparticles

Magdalena Ziąbka[1]*, Michał Dziadek[2]

[1]AGH University of Science And Technology, Faculty of Materials Science and Ceramics, Department of Ceramics and Refractories, Krakow 30-059, Poland
[2]AGH University of Science and Technology, Faculty of Materials Science and Ceramics, Department of Glass Technology and Amorphous Coatings, Krakow 30-059, Poland
*Correspondence: e-mail: ziabka@agh.edu.pl

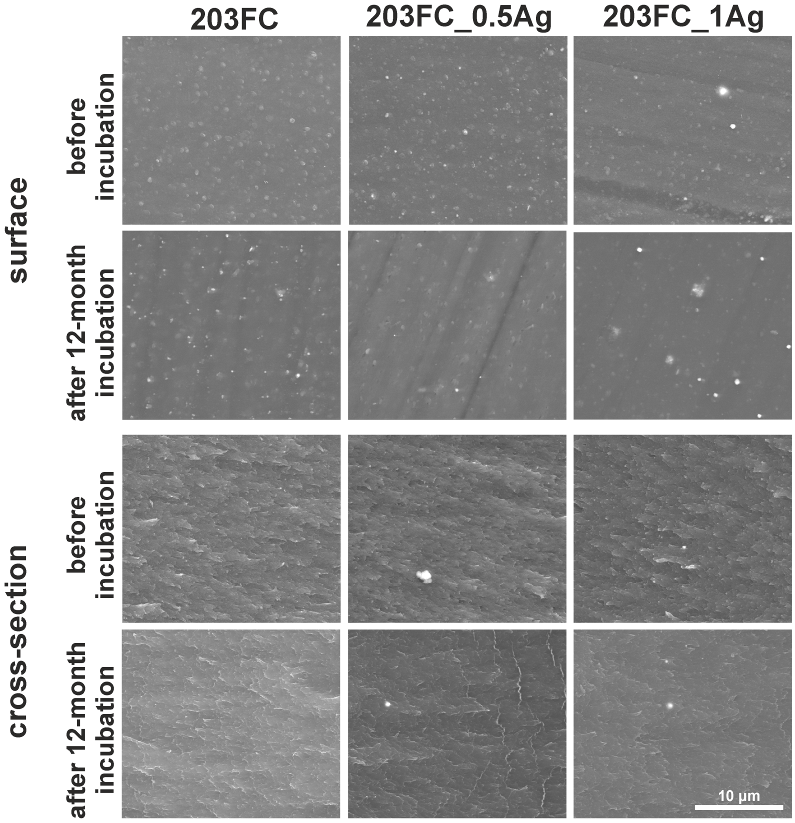

Figure S1. SEM images of surface and cross-section of the 203FC-based materials before and after 12-month incubation in deionized water.

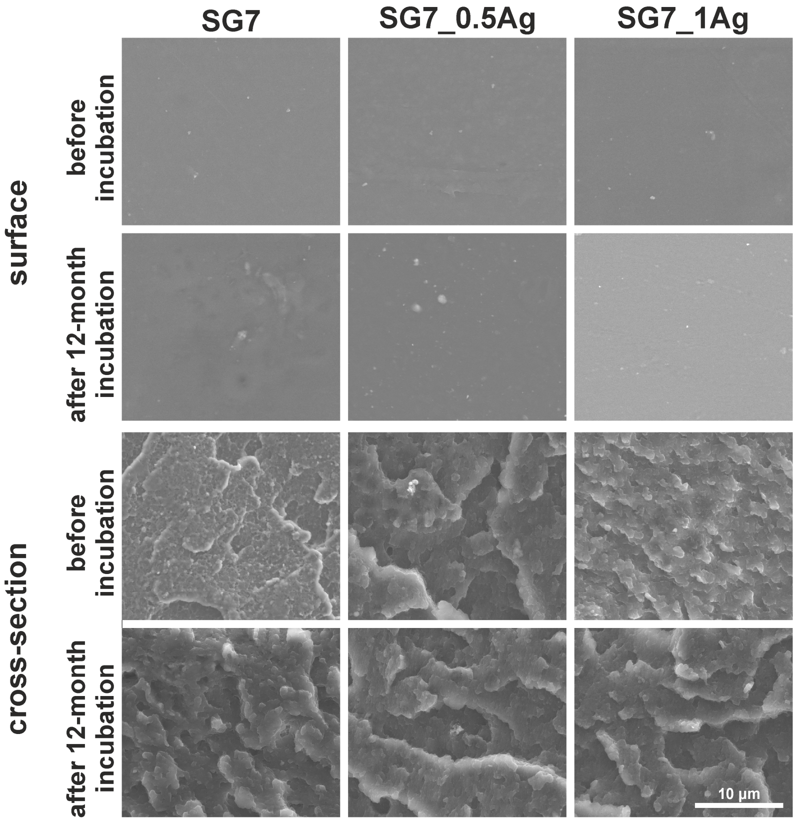

Figure S2. SEM images of surface and cross-section of the SG7-based materials before and 12-month incubation in deionized water.

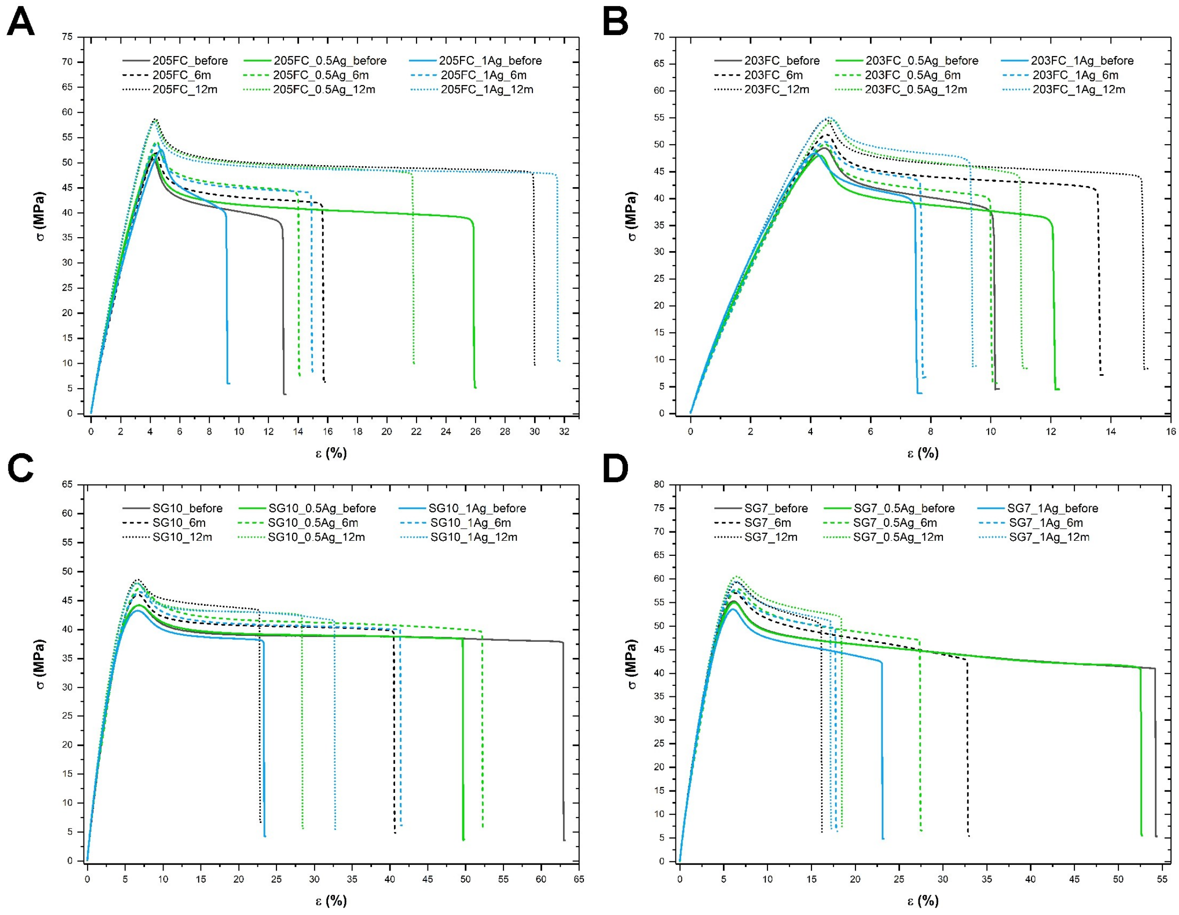

Figure S3. Tensile stress-strain curve of the 205FC- (A), 203FC- (B), SG10- (C), and SG7-based (D) materials before and after 6-month and 12-month incubation in deionized water.
